# 1543. Validation of a Nitrocefin-based Rapid Test for the Detection of the Cefazolin Inoculum Effect in Pediatric Patients with Methicillin-Susceptible *Staphylococcus aureus* Acute Osteomyelitis

**DOI:** 10.1093/ofid/ofac492.098

**Published:** 2022-12-15

**Authors:** Sara I Gomez Villegas, Sandra Rincon, Lina Carvajal, Lauren M Sommer, Diana Panesso Botero, Maria Reyes, Rafael Rios, An Dinh, Lorena Diaz, Sheldon L Kaplan, Jinnethe Reyes, Anthony R Flores, Jonathon C McNeil, Cesar A Arias

**Affiliations:** Brigham and Women’s Hospital, Houston, TX; Universidad El Bosque, Bogota, Distrito Capital de Bogota, Colombia; Universidad El Bosque, Bogota, Distrito Capital de Bogota, Colombia; Baylor College of Medicine, Houston, Texas; Houston Methodist Research Institute, Houston, Texas; Houston Methodist Research Institute, Houston, Texas; Universidad El Bosque, Bogota, Distrito Capital de Bogota, Colombia; Houston methodist research institute, Houston, Texas; Universidad El Bosque, Bogota, Distrito Capital de Bogota, Colombia; Baylor College of Medicine, Houston, Texas; Molecular Genetics and Antimicrobial Resistance Unit, Universidad El Bosque, Bogota, Distrito Capital de Bogota, Colombia; University of Texas Health Sciences at Houston, Houston, Texas; Baylor College of Medicine, Houston, Texas; Houston Methodist Hospital and Weill Cornell Medical College, Houston, TX

## Abstract

**Background:**

The cefazolin (Cz) inoculum effect (CzIE) defined as a Cz MIC ≥ 16 µg/mL at high inoculum (10^7^ CFU/mL) in MSSA isolates has been associated with poor outcomes in adult patients with bacteremia. Recently, it was associated with higher rates of progression to chronic osteomyelitis in pediatric patients with acute MSSA osteomyelitis, independent of the treatment choice. Thus, its detection could be important for management of certain MSSA infections. Broth microdilution at high inoculum is the gold standard for the detection of the CzIE. Yet, this method is lengthy (3–5 days), cumbersome, and expensive. We developed a nitrocefin-based rapid test (∼3h) which can identify the CzIE among MSSA isolates and that can be incorporated in the laboratory workflow. Previously, this rapid test was validated in MSSA isolates from adult patients with bacteremia. Here, we aimed to validate it in isolates from pediatric patients with acute MSSA osteomyelitis

**Methods:**

We included 206 MSSA isolates from the same number pediatric patients from Houston, TX (2011–2018). All patients were diagnosed with acute MSSA osteomyelitis. The CzIE was determined using broth microdilution at high inoculum with a cutoff of Cz MIC ≥ 16 µg/mL. Whole genome sequence analysis was performed using Illumina Hi-Seq. BlaZ type was determined according to predicted BlaZ residues at position 119 and 207. The nitrocefin rapid test was performed following the published protocol (Rincon et al., *JCM*, 2021). Performance metrics were calculated for the complete data set and for specific BlaZ types.

**Results:**

The prevalence of the CzIE was 33%. Compared to the gold standard, the nitrocefin rapid test had a sensitivity of 81% and a specificity of 93% (Table 1). The false negative and false positive rate of 9.2% and 13.8%, respectively, with an overall accuracy of 89%. There were no false positive results among *blaZ* negative strains. Among type A BlaZ MSSA, the sensitivity was 96.7%, and the specificity was 80%, while in type C BlaZ, they were 70.5% and 92.8%, respectively.

Performance metrics of the Nitrocefin-based Rapid Test in 206 MSSA isolates from pediatric patients with acute MSSA osteomyelitis compared to the Gold Standard of Broth Microdilution at high Inoculum.

**Conclusion:**

The nitrocefin-based rapid test was able to correctly detect the CzIE in isolates from pediatric patients with MSSA acute osteomyelitis, with an overall accuracy of 89%. Implementation of this test may contribute to therapeutic decisions in deep-seated MSSA infections.

**Disclosures:**

**Sheldon L. Kaplan, MD**, MeMed: Grant/Research Support|Pfizer: Grant/Research Support **Jonathon C. McNeil, MD**, Agency for Healthcare Research and Quality: Grant/Research Support|Allergan: Provided reagents for unrelated research|Nabriva: Site investigator for multicenter clinical trial **Cesar A. Arias, MD, PhD**, Entasis: Grant/Research Support|MeMed Diagnostics Ltd: Grant/Research Support|Merck: Grant/Research Support.

**Table 1.**
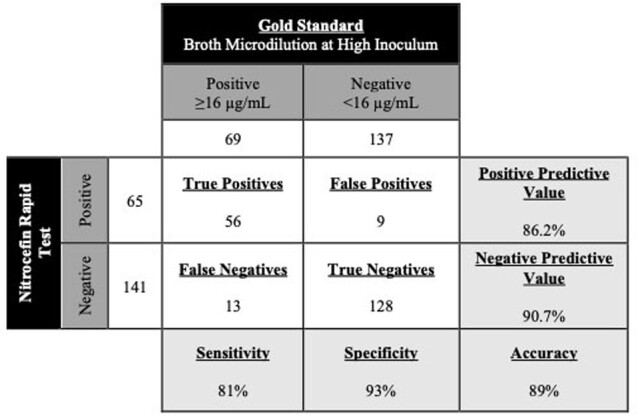
Performance Metrics of the Nitrocefin-based Rapid test among MSSA causing acute osteomyelitis.

